# Case report: Variability in clinical features as a potential pitfall for the diagnosis of Barth syndrome

**DOI:** 10.3389/fped.2023.1250772

**Published:** 2023-08-16

**Authors:** Nicola Tovaglieri, Silvia Russo, Emanuele Micaglio, Angela Corcelli, Simona Lobasso

**Affiliations:** ^1^ Department of Pediatrics, Niguarda Hospital, Milan, Italy; ^2^ Department of Translational Biomedicine and Neuroscience, University of Bari Aldo Moro, Bari, Italy; ^3^ Department of Arrhythmology and Clinical Electrophysiology, Institute of Molecular and Translational Cardiology (IMTC), IRCCS Policlinic San Donato, Milan, Italy

**Keywords:** rare X-linked disease, TAFAZZIN, cardiolipin remodeling, cardiomyopathy, neutropenia

## Abstract

**Background:**

Barth syndrome is a rare genetic disease characterized by cardiomyopathy, skeletal muscle weakness, neutropenia, growth retardation and organic aciduria. This variable phenotype is caused by pathogenic hemizygous variants of the *TAFAZZIN* gene on the X chromosome, which impair metabolism of the mitochondrial phospholipid cardiolipin. Although most patients are usually diagnosed in the first years of life, the extremely variable clinical picture and the wide range of clinical presentations may both delay diagnosis. This is the case reported here of a man affected with severe neutropenia, who was not diagnosed with Barth syndrome until adulthood.

**Case presentation:**

We describe herein a family case, specifically two Caucasian male cousins sharing the same mutation in the *TAFAZZIN* gene with a wide phenotypic variability: an infant who was early diagnosed with Barth syndrome due to heart failure, and his maternal cousin with milder and extremely different clinical features who has received the same diagnosis only at 33 years of age.

**Conclusions:**

Our report supports the underestimation of the prevalence of Barth syndrome, which should be always considered in the differential diagnosis of male patients with recurrent neutropenia with or without signs and symptoms of cardiomyopathy.

## Introduction

Barth syndrome (BTHS, MIM 302060) is a rare X-linked disorder associated with a wide spectrum of effects, particularly including early-onset cardiomyopathy, skeletal muscle myopathy, neutropenia, growth delay, organic aciduria and variable mitochondrial dysfunction (1–4). Loss-of-function mutations in the *TAFAZZIN* gene, located on chromosome Xq28.12, cause defective acyl-chain remodeling of the phospholipid cardiolipin (CL), resulting in mitochondrial bioenergetics and metabolic abnormalities in patient tissues (5–8). CL is a dimeric phospholipid with four acyl chains localized almost exclusively in the inner mitochondrial membrane, where it plays a key role in maintaining both structural and functional integrity of many mitochondrial proteins, including respiratory chain protein complexes and their assembly into supercomplexes (9–12). Loss of function of the protein tafazzin leads to an altered CL transacylation, resulting in depletion of remodeled CL forms (mainly tetra-linoleoyl CL in the heart) with accumulation of immature CL forms (containing more saturated acyl-chains) and monolysocardiolipin (MLCL), a phospholipid containing three acyl chains which is practically absent in healthy mitochondria. Therefore, alterations of CL remodeling causes a dramatic increase of the MLCL/CL ratio in BTHS mitochondria (13).

Diagnosis of BTHS can be tricky because the clinical and biochemical features of the syndrome are highly variable and may differ between affected individuals from the same family and within a patient over time (2). Many patients with BTHS have mildly to severely elevated levels of 3-methylglutaconic acid (3-MGCA) on quantitative analysis of urinary organic acids, so that the disorder is also referred to “type II 3-methylglutaconic aciduria” (2). However, the urinary 3-MGCA levels can be elevated in a number of other metabolic disorders (14), so this test shows poor diagnostic specificity.

In contrast, the measurement of the MLCL/CL ratio is the only biochemical test characterized by high diagnostic sensitivity and specificity and it can be performed in bloodspots, fibroblasts and nucleated blood cells (15–17). The elevated MLCL/CL ratio is considered the biochemical hallmark of BTHS. Molecular genetic testing for mutations in the *TAFAZZIN* gene is also used for the diagnosis of BTHS. More than 120 pathogenic mutations spanning all 11 exons and introns of the *TAFAZZIN* gene are known (4). Nevertheless, no correlation between genotype/phenotype in BTHS has been demonstrated to date.

## Case description

Here we describe the wide clinical variability in age of presentation, severity, and type of symptoms of two male relatives with the same genetic mutation in the *TAFAZZIN* gene: a child with classic cardiac features (Patient 1) and an oldest patient presenting mainly neutropenia without heart failure (Patient 2).

**Figure 1:**
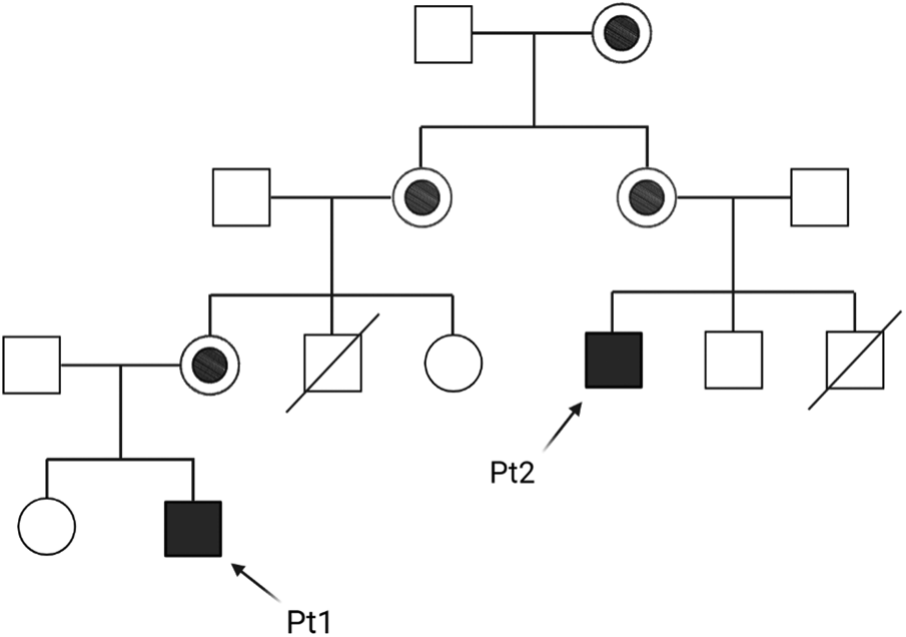
Family pedigree of patients 1 and 2 (Pt1 and Pt2).

Patients 1 and 2 belong to the same family, whose pedigree is shown in Figure 1.

We conducted this case study to aid in the diagnosis of BTHS and to highlight the need for a re-evaluation of diagnostic approaches, particularly with reference to patterns of neutropenia in male patients.

## Diagnostic assessment and details

Patient 1 is a Caucasian child born full-term and with normal growth parameters after an uneventful pregnancy; as his maternal uncle died for complications of dilated cardiomyopathy (DCM) at age 7 months, the patient's cardiac function was evaluated by transthoracic echocardiography at age 2 months. Cardiac examinations revealed DCM with hypertrophied and hypertrabeculated walls, a mild-moderate mitral insufficiency and impaired left ventricular systolic function with an ejection fraction (EF) of about 38%.

Based on these findings, BTHS was clinically suspected and diagnosed by the genetic evaluation which identified a hemizygous DNA mutation (c.777 + 1G > A) in intron 10 of the *TAFAZZIN* gene, which is a splice donor variant classified as likely pathogenic (18). The patient started pharmacological treatments for heart failure with paediatric dosage of beta-blockers, ACE inhibitors, diuretics, and ivabradine.

At age 3, his EF was about 45%, while the Holter QTc was 448 msec with abnormal ventricular repolarization. His therapy was modified by quitting the diuretic. From then on, the EF has been around 52%–55% and the Holter QTc normal; however, left ventricular noncompaction (LVNC), a cardiomyopathy characterized by loose and trabeculated myocardium that is often associated with BTHS (2, 3), was observed.

Currently, the patient is 6 years old. His last cardiac evaluation showed increased wall thicknesses on the inferior wall and on all mid-apical segments (baseline IVS 5.5 mm, PW 8 mm), accentuation of the trabecular meshwork, a slight reduction of systolic function overall (LVEF 47%–48%) together with mild reduction of the mitral annular plane systolic excursion (MAPSE 9 mm).

Regarding neutropenia, another pathological condition associated with BTHS (2, 3), the patient showed a normal neutrophil count with episodic moderate neutropenia. At age 2, during an infection episode accompanied by severe neutropenia (neutrophil count 350/µl), therapy with Granulocyte Colony-Stimulating Factor (G-CSF) was started. However, the patient had remarkable neutrophilia, so therapy was stopped a few months later, and he never again experienced severe bacterial infections.

As regards metabolic screening, the first evaluation of plasmatic amino acids and organic urinary acids was performed at age 2, when the baby came to our attention, and showed augmented excretion of 3-MGCA. The patient was monitored for metabolic alterations from then on, and low citrulline and arginine levels were detected during the most recent follow-up, which have been corrected by increasing supplementation therapy (citrulline, arginine and vitamin B12).

No significant episodes of hypoglycaemia occurred, except for at 3 years of age, during a viral gastroenteritis. He is now under therapy with Glycosade, a slow-release starch to keep blood sugar levels consistent and prevent hypoglycaemia.

Neither skeletal muscle weakness nor growth retardation have been observed in Patient 1 until now.

Patient 2 is a maternal cousin of Patient 1 (see Figure 1), a 33-year-old Caucasian male with recurrent severe neutropenia since early childhood, who was not diagnosed with BTHS until adulthood. This patient had two brothers, the first one died due to congenital cardiomyopathy at age 1 month, while the second one is in good health (but without genetic investigation yet to date). Cardiac function parameters have always been normal throughout his life until now. However, neutropenia was observed at birth after an acute urinary tract infection, and then the patient was hospitalized at 1 year of age for septic shock due to a severe *Pseudomonas aeruginosa* infection. This event was followed by frequent and important infectious episodes (mainly either middle ear or inner ear infections) with hospitalization during the first years of life. After repeated neutrophil count examinations, which often indicated a value <500/µl, the patient started therapy with 4 µg/Kg G-CSF on alternate days at 4 years of age, with good recovery of neutrophil count and no other infectious episodes.

At age 11, after discontinuation of G-CSF therapy, the patient was hospitalized again for febrile gastroenteritis with severe dehydration. During this time, he suffered from cardiac arrest and then septic shock after surgical hemi-colectomy for two colic perforations. After this event, the G-CSF therapy was reinstated with a contextual normalization of the neutrophil count and only sporadic leucocytosis.

During his adolescence, the patient showed delayed growth in stature and weight and a bone mineral density that was lower than expected for gender and age (calculated T-score—2.9 at spinal level at 15 years of age). This finding is indicative for the presence of moderate osteopenia. Because of the associated presence of a hypovitaminosis D condition, supplemental therapy with calcifediol was introduced.

At age 23, the T-score at spinal level improved slightly to −2.2, but vertebral fractures (at D11, D12, and L1) were observed. For this reason, the patient was also treated with bisphosphonates by infusion.

At age 25, the patient was evaluated by a geneticist for hematopoietic system disorders by DNA sequencing and mutation analysis in a panel of candidate genes, including *ELANE*, *HAX1*, *G6PC3*, and *WAS*. However, the sequences were found to be normal, and no signs of immunodeficiency nor Wiskott-Aldrich syndrome has been noted after a careful genetic counselling. The patient had electrocardiogram and echocardiogram for physical activity normal.

At age 33, given his clinical history and over all the familiarity, the diagnosis of BTHS was finally made, 5 years later than the cousin's diagnosis. First, an elevated MLCL/CL ratio was detected in the leukocytes by MALDI-TOF/MS (17, 19). Finally, DNA sequencing of the *TAFAZZIN* gene showed that Patient 2 shared the same genetic mutation with his cousin, Patient 1.

After diagnosis, cardiac evaluation of Patient 2 showed a mild EF reduction that did not require therapy. Patient 2 waits for the evaluation of the amino acids to possibly fill in the missing ones.

Table 1 summarises the most important clinical, biochemical, and genetic findings of the two BTHS patients, and shows a considerable phenotypic variability.

**Table 1:** Most important clinical, biochemical and genetic findings in the two patients.

Parameters	Patient 1	Patient 2
Age at diagnosis (years)	1	33
Mutation	TAFAZZIN (C.777 + 1G > A)	TAFAZZIN (C.777 + 1G > A)
Increased MLCL/CL ratio	Yes	Yes
3-MGCA-uria	Yes	N/A
Cardiomyopathy	Yes	No
Neutropenia	Yes (moderate/episodic)	Yes (severe/recurrent)
Skeletal myopathy	No	No
Osteopenia	No	Yes
Growth delay	No	Yes
Facial dysmorphia	Yes	Yes (mild)
Recurrent infections	No	Yes

## Discussion

BTHS is commonly classified as a paediatric disorder because most patients are diagnosed in the first years of life. The estimated prevalence of BTHS is 1 case per million males, with about 15 patients currently known in Italy and 250 worldwide, although it is generally accepted that the disease is underdiagnosed (20).

There are increasing reports of patients diagnosed with BTHS in adulthood (21, 22), suggesting that knowledge about this rare disorder has been growing among physicians in recent years. In light of the case study here reported, it should be considered that many more adult patients with idiopathic cardiomyopathy or recurrent neutropenia might be possible unidentified diagnoses of BTHS.

Although heterogeneous clinical features and intrafamilial variability have been previously reported in affected members harbouring the same genetic mutation in *TAFAZZIN* (4, 23), the significant variability characterizing the two cousins of this family is striking. BTHS patients without paediatric cardiac symptoms are rare (23); indeed, cardiomyopathy and heart failure are the primary reasons for diagnosis in infantile period and death (2, 4). The most common cardiomyopathies described in BTHS patients are DCM and LVNC (2, 4).

In contrast to Patient 1, Patient 2 of this study did not show significant cardiac disease as a child, but frequent infectious events due to severe and recurrent neutropenia were the main features of his disorder. Neutropenia (absolute neutrophil count less than 1,500/mcl) is one of the most frequently observed findings in BTHS patients and one of the earliest manifestations of the disease, which can range from absent to severe, intermittent, or recurrent (2, 24). The aetiology of neutropenia in BTHS is not yet understood (24).

As described above, Patients 1 and 2 have a different clinical presentation, although the same genetic mutation in the TAFAZZIN gene and similar increased levels of MLCL/CL ratio have been detected.

Of course, it can be also considered that many clinical parameters are different because of the vast age difference in patients. Of note, both patients presented with typical cranial-facial appearance, characterized by full cheeks, deep set eyes and prominent ears (in some patients with fleshy lobes) (2, 23).

To date, it remains unclear whether additional modifying genetic factors might contribute to the differential and pleiotropic phenotypic expression of BTHS patients. A recent study has demonstrated that genetic background in a mouse BTHS model powerfully impacted phenotypic expression of tafazzin loss-of-function possibly altering mitochondrial quality control (25). These data together with many reports describing no correlation between the type of mutation and clinical presentation in BTHS patients clearly indicate the existence of additional modifying factors that act as key determinants of pathogenesis of the syndrome (26).

In addition, splicing diversity may be responsible for the variability of BTHS phenotypes. Recently a case of infantile BTHS patient with severe heart failure and LVNC has been reported, in which amino acid substitution associated with a missense variant in TAFAZZIN gene was not sufficient to explain the syndrome severity, but it exhibited various splicing variants and contributed to the worse clinical course (27). These findings suggest that it is important to determine whether missense variants in TAFAZZIN are related to splicing abnormalities in BTHS.

Patient 2 here described did not receive the correct diagnosis of BTHS until adulthood and was probably diagnosed only due to his family history. As with other rare genetic diseases, it is important to identify the underlying genetic pathology in patients, although there is no specific therapy for BTHS to date. Early diagnosis is critical to allow rapid intervention for heart failure and antibiotic treatment for bacterial infections, thereby improving patients' quality of life. In addition, treatment of such patients requires a multidisciplinary team approach due to the variety of organ systems that could be involved.

In conclusion, the study here reported corroborates the underestimation of the prevalence of this rare genetic disease, which should always be considered in the differential diagnosis of male patients with unexplained neutropenia. Therefore, we suggest including *TAFAZZIN* mutation test in the next generation sequencing analysis designed to investigate idiopathic neutropenia.

## Data Availability

The original contributions presented in the study are included in the article, further inquiries can be directed to the corresponding author.
